# Bridging the knowledge gap! Health outcomes in informal e-waste workers

**DOI:** 10.1186/s12995-024-00410-z

**Published:** 2024-04-15

**Authors:** Béla Eckhardt, Andrea Kaifie

**Affiliations:** 1 Institute for Occupational, Social, and Environmental Medicine, Medical Faculty, RWTH Aachen University, Pauwelsstrasse 30, 52074 Aachen, Germany; 2 Institute and Outpatient Unit for Occupational Social and Environmental Medicine, Medical Faculty, FAU Erlangen-Nuremberg, Germany

**Keywords:** WEE, Disease/disorders, Morbidity, Symptom burden, Work

## Abstract

**Background:**

Although several studies analyzed the impact of e-waste recycling on human health, most publications did not differ between e-waste workers and bystanders, such as residents. This could lead to an underestimation of health effects in workers. In addition, frequently reported surrogate findings do not properly reflect clinical significant health outcomes. The aim of this review was to analyze the direct health effects of informal e-waste recycling in informal e-waste workers.

**Methods:**

According to PRISMA guidelines, we systematically searched 3 databases (Embase®, PubMed®, Web of Science) for studies from low- and middle-income countries published in German or English between 1980 and 1 November 2021. Of the 2613 hits, 26 studies (cross-sectional, longitudinal and case-control studies) met the specified criteria and were included. We categorized the results into hormonal, respiratory, renal, cardiovascular, musculoskeletal health and general symptoms in informal e-waste workers.

**Results:**

Exposure to e-waste was associated with altered lipid metabolism, thyroid hormonal imbalances, impaired fertility, renal dysfunction, increased prevalence of respiratory symptoms, asthma, cardiac arrhythmias, hypertension, musculoskeletal pain, injuries in up to 89% and skin disorders in up to 87.5–100% of e-waste workers.

**Conclusion:**

Due to inconsistent findings, weak associations or poor study quality, it has rarely been possible to establish a causal relationship between informal e-waste work and health effects, except for injuries or skin conditions. Besides high-quality studies, a collective national and international political focus on e-waste disposal is needed.

**Supplementary Information:**

The online version contains supplementary material available at 10.1186/s12995-024-00410-z.

## Introduction

The increasing amount of electronic waste is a global problem [1]. It is considered to be the fastest growing waste-stream in the European Union (EU) driven by the rapid increase in the use and disposal of electronic devices [2, 3]. When electrical and electronic equipment (EEE) is disposed with no intention of reuse, it becomes e-waste [4].

According to the United Nations (UN) Global E-waste Monitor, the global e-waste increased from 44.4 million metric tons (Mt) in 2014 to 53.6 million Mt in 2019. This number is expected to rise to 74.7 million metric tons by 2030. Less than 20% of the global WEEE were documented to be properly recycled and collected [4]. The large undocumented part of e-waste ends up in landfills, is incinerated or illegally shipped to low- and middle-income countries where regulations may be non-existent or inadequate and the processing is performed in an inferior way [1, 5–7]. High-volume informal recycling and processing of WEE have been reported in several countries, including China, Ghana, Nigeria, India, Thailand, Pakistan and Vietnam [8–10]. Since e-waste contains a significant number of materials of value, the recycling process holds an economic opportunity for developing countries. Socially disadvantaged populations depend on trade, repair, and recuperation of materials from e-waste as a source of income [8, 11], and many e-waste workers are not aware of the hazards and potential health risks associated with e-waste recycling [7, 9].

The informal recycling process includes collecting, manual dismantling, separation, and mechanical pre-treatment of e-waste, open burning, pyrometallurgical processes like refining, smelting, combustion and incineration using high temperatures as well as acid baths and cyanide salt leaching [12, 13]. The severity of toxin release and exposure depends on the respective work focus of the workers during the recycling process [13].

In a systematic review Grant et al. examined the health consequences of general exposure to e-waste. They discovered associations between exposure to e-waste and a number of harmful health effects, including poor birth outcomes, delayed neurological and behavioural development, (inconsistent) thyroid function changes and a higher chance of developing chronic diseases in later life [9]. According to the updated version from Parvez et al., possible connection between long-term exposure to e-waste and DNA damages, telomere shortening and alterations in immune system function were found [14]. However, in these systematic reviews no differentiation has been made between the group of e-waste workers and bystanders, including residents in e-waste recycling areas. It can be assumed that e-waste workers who are directly involved in the recycling process are far more exposed to hazardous substances compared to bystanders or residents, who may only be indirectly exposed through the release of pollutants into the surrounding air, water, food, or soil. This may lead to a significant underestimation of the health impacts of informal e-waste recycling on e-waste workers.

Therefore, in this review we particularly focused on e-waste workers involved in informal e-waste recycling activities in order to assess and summarize health effects of informal e-waste recycling [15].

## Materials and methods

### Protocol and registration

The systematic review on health effects in e-waste workers was carried out using the *Preferred Reporting Items for Systematic Reviews and Meta-Analyses* (PRISMA) guidelines [15]. We prepared a systematic study protocol which we submitted to the *International prospective register of systematic reviews* (PROSPERO) by the university of York [16]. The review was accepted and registered on 21st January 2022 under the record number CRD42022299134. It can be found under https://www.crd.york.ac.uk/prospero/display_record.php?ID=CRD42022299134.

### Search strategy and eligibility criteria

The research question as well as the systematic search strategy was designed following the PECO scheme (Population, Exposure, Comparison and Outcome)(Table 1). To investigate the health effects, symptoms and diseases associated with working in the informal e-waste sector, we exclusively included workers in the informal e-waste recycling sector with occupational exposure during the recycling process. Furthermore, the outcome had to be a clinical symptom or a disease. Surrogate outcomes, such as oxidative stress or DNA damage that does not necessarily lead to a clinical effect were excluded. Residents, bystanders, people with no connection to e-waste recycling were strictly excluded.

**Table 1:** Inclusion and exclusion criteria

Peco Sceme	Inclusion Criteria	Exclusion Criteria
Population (P)	Workers (including adults, adolescents (age under 18 years), children) in the informal e-waste recycling sector in middle- and low-income countries with an occupational exposure to e-waste	Adults, children or adolescents with no connection to e-waste recycling, residents and bystanders with only environmental exposure, as well as workers from high income countries and/or from the formal e-waste sector
*Exposure (E)*	Exposure to hazardous substances as well as mechanical, ergonomic, psychological, and physical hazards related to work in the informal e-waste recycling sector	Only environmental exposure, bystander exposure
Comparator(s)/Control (if available)(C)	Workers (incl. Adults, children and adolescents) in the informal e-waste recycling sector without occupational exposure to e-waste	Adults, (preschool) children, adolescents, bystanders, people involved in informal e-waste recycling activities
Outcome (O)	Disorders or diseases, symptoms and further health effects associated with or caused by informal e-waste recycling as well as its influence on long time health, morbidity, mortality	No direct effects on health, effects without a clinical correlate (such as oxidative stress, DNA damage

We included prospective, observational, cross-sectional or case-control-studies, (systematic) reviews and Meta-analyses in low- and middle-income countries in the informal e-waste recycling sector. Case reports, methodological or interventional studies and all other kinds of studies were excluded, as well as studies in high income countries, with a context of formal e-waste recycling or studies that do not match the above-mentioned criteria. Studies published in German or English between 1980 and 01.11.2021 were included.

We searched three electronic databases, Embase®, PubMed® and Web of Science. Additional further studies were included out of the references of the screened papers. To ensure that all potentially matching results were included, the keywords were merged using the Boolean operators AND (to combine the categories) and OR (to combine the keywords in a category) (supplemental section S1).

The research was conducted on 25th November 2021 and a total of 2613 hits could be found in all three databases.

### Screening process

The search results were extracted into an Excel spreadsheet and duplicates were removed. The articles were then systematically and independently screened by the two authors. First, a title screening and abstract screening with a selection process was carried out. A full text screening for the remaining articles followed subsequently. After each screening step, the selected studies were compared. In case of disagreement, the studies in question were checked again for the previously set criteria and discussed until a consensus was found. Each exclusion of a study was documented. The reasons for the exclusion were documented during the full-text screening.

### Data collection (quantitative assessment)

A table containing all relevant information from each study was created. This data extraction was categorized into author and year, study design, setting and time, participants, exposure, measurements, outcome and if available effect parameters for each study. If presented, statistical mean, standard deviations, and *p*-value (‘bold’ if significant) were documented. Depending on the data situation and given statistics, socio-demographic information was extracted. Special attention was paid to the statistical differentiation of the e-waste workers from (if given) comparison groups (such as bystanders or residents).

### Bias assessment (qualitative assessment)

The methodological qualitative assessment for each study was conducted independently by two authors. Subsequently, disagreements were reviewed and discussed until a common consensus was found. The bias assessment was carried out for each study using a checklist for measuring study quality initially published by Downs and Black [17]. The bias risk of the following categories was assessed: Internal validity bias (such as blinding of participants, data dredging or outcome measures), internal validity confounder (such as recruitment, losses of follow-up or randomization), performance bias, detection bias (like recall or information bias), attrition bias and reporting bias. In addition, a category with ‘other bias’ was recorded, where individually varying biases of the respective studies were recorded (such as selection bias and special features). The answer options were yes, no, and not applicable (n.a.). Each bias was given a risk rating that corresponded to the categories (mentioned above): low risk when all questions referring to a possible existing bias were classified as low risk, high risk if at least one question indicated a high risk for a possible existing bias, and when at least one question was answered with an undetermined response an unclear risk was assumed (Table S2).

Since most of the study designs were cross-sectional studies, no ‘follow-up procedure’ had been applied within these study designs, as well as no ‘losses of follow-up mentioned’, which we then labelled as not applicable (n.a.). Only one Study had a longitudinal study design with a short follow up procedure [18]. Since no interventions were carried out in most of the studies, the questions about blinding of staff and participants could mostly be answered in the negative or as n.a.. The questions about confounders ‘random sequence generation’ and ‘allocation concealment’ were also classified as n.a. N.a. was not taken into consideration in the risk of bias assessment (Table S2).

### Study selection

The systematic literature searches in the three databases Pubmed, Embase and Web of Science resulted in 2613 hits. After removing all duplicates, all studies that did not meet the eligibility criteria were sorted out during title and abstract screening. The remaining articles, as well as one additional study [19] handpicked from a review, were included into the full-text screening.

**Fig. 1:**
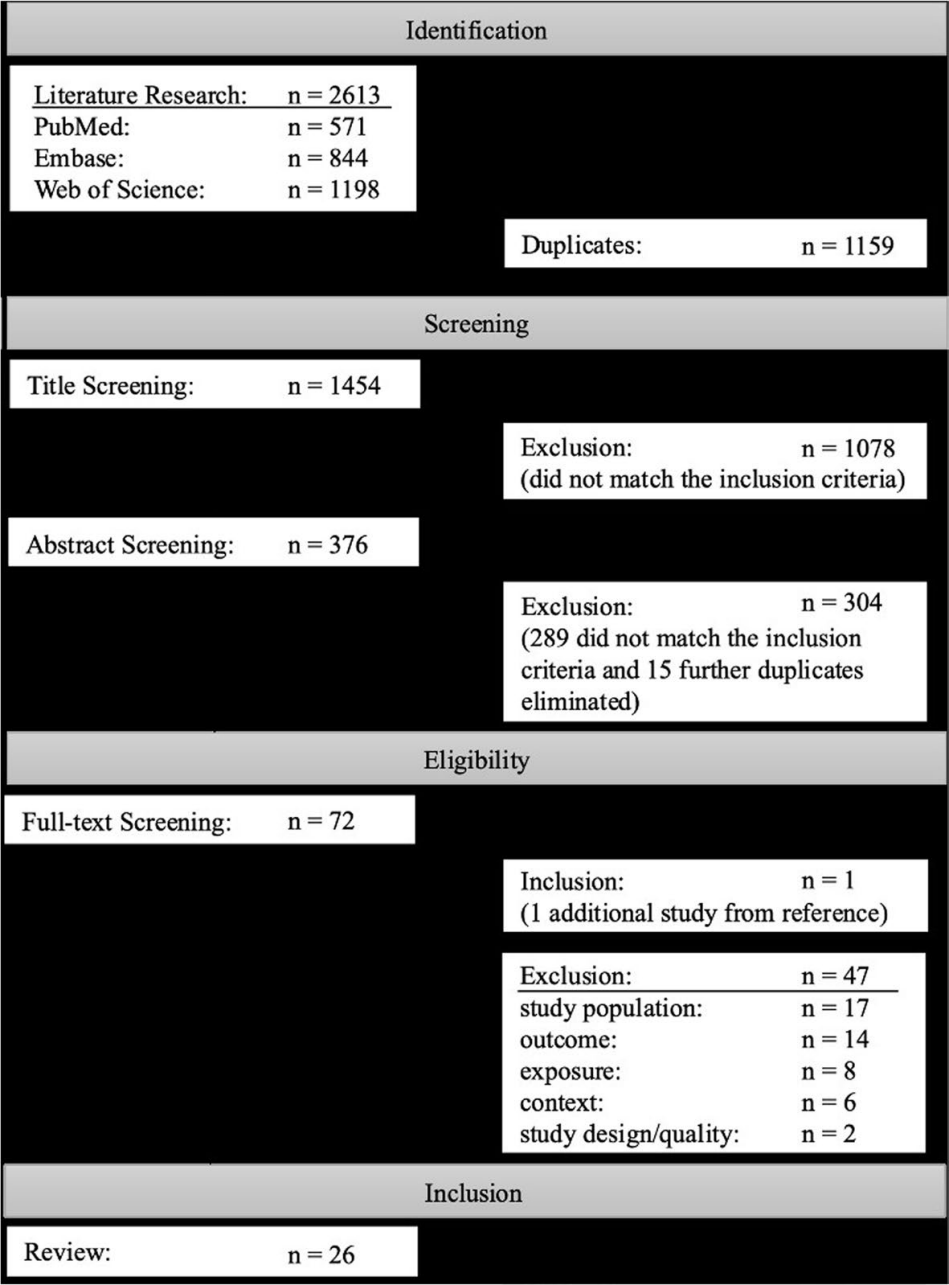
Flow chart of included studies during the screening process

26 studies met the eligibility criteria and were included in this systematic review [18–43], see Fig. 1.

## Results

### Literature research and screening process

The 26 included articles consisted of 23 cross-sectional studies [2–24], 1 scoping review [34], 1 nested case-control study [33], 1 longitudinal cohort study [18] and comprised a publication period from 2008 to 2021. Geographically, the included studies have been conducted in the following regions:

*Africa*: Nigeria [19, 32, 34, 36], Ghana [18, 20–26, 30, 31, 34],

*Asia*: Vietnam [28, 29], China [38, 39, 41–43], Thailand [27, 33, 35, 37], India [34],

*South America*: Chile [40].

We often found overlaps of health outcomes across categories. Therefore, we assigned each study to the category most likely to apply and the outcomes of the study to the relevant section. We grouped the studies into 7 categories depending on the mainly investigated organ system/health effect to provide a better overview of the results.

#### Hormonal health

A total of 9 cross-sectional studies on the potential effects of exposure to e-waste and hormonal health were identified (Table 2). In 2014 and 2015 Eguchi et al. analysed concentrations of thyroid hormones (THs) in serum samples from e-waste workers in Vietnam and found FT3, TT3 [28, 29] and TT4 concentrations [29] to be significantly lower than the samples from the control group living at a rural site [28, 29]. The multiple linear regression showed a significant association between specific circulating TH levels and organic contaminants (OC) [43].

**Table 2:** Studies concerning hormonal health outcomes

	Study design, exposure, setting, time	Population/ participants	Measurements, Examination	Health outcome
Hormonal health				
(incl. Thyroid function, lipid metabolism and fertility/reproductive systems)				
Eguchi et al. [2015]	Cross-sectional: exposed e-waste worker (EWW) vs unexposed control group (CG), Vietnam, 2010–2011	Overall *n* = 111 participants 77 EWW (45 females, 32 males) 34 residents as CG (22 females, 12 males)	Thyroid hormones (TH) in serum samples Personal interview incl. Demographic-, health- and diet information	TH-concentrations were within normal limits, although TT4, TT3 and FT3 concentrations in serum samples from the e-waste recycling site (Bui Dau) were significantly lower than at the reference site (Duong Quang). Significant gender differences in the TH levels with higher levels of TT4, TT3 in women were found.
Zheng et al. [2017]	Cross-sectional: exposed e-waste worker (EWW), South China, 2011	79 EWW (36 females, 33 males)	Circulating THs (incl. TT4, FT4, TT3, FT3, TSH) in serum samples Physical examination & questionnaire incl. Health, occupational & demographic information	TH concentrations in EWW were generally within the population reference ranges and no significant gender differences in the TH levels, except for FT3 were found. Multiple linear regression coefficients for a significant association between specific circulating TH levels and OCs were found for TT3 & BDE47**,** TT3 & BDE85.
Wang, H et al. [2010]	Cross-sectional: exposed e-waste worker (EWW) vs non-occupational exposed group/residents (R) vs unexposed control group (CG), China, November–December 2008	Overall *n* = 442 236 EWW from 3 E-waste sites 89 residents (R) 117 residents as CG	THs (incl. TT4, FT4, TT3, FT3, TSH) in serum samples Questionnaire incl. Health- and dietary information, demographic- and occupational history)	Residents and EWW had significantly lower serum T3, fT3, fT4 levels than the control group. TSH concentrations were significantly lower within the EWW compared to controls.
Eguchi, A et al. [2014]	Cross-sectional: exposed e-waste worker (EWW) vs unexposed control group (CG), Vietnam, January 2010–January 2011	Overall *n* = 131 participants 83 EWW (48 females (2 pregnant), 35 males) 48 unexposed as CG (33 females (2 pregnant), 15 males)	THs (incl. TT4, FT4, TT3, FT3, TSH) in serum samples Interview incl. Demographic-, health- and diet information, pregnancy status	Concentrations of TT3 and FT3 were significantly lower in EWW than in the CG.
Yuan et al. [2008]	Cross-sectional: exposed e-waste worker (EWW) vs unexposed control group (CG), China, study period not reported	Overall *n* = 49 participants 23 EWW (7 females, 16 males) 26 farmers as CG (11 females, 15 males)	TSH levels in serum samples Questionnaire incl. Personal medical history, smoking history, alcohol consumption, occupational history	Concentrations of TSHs in EWW were significantly higher than in the CG. A multivariate logistic regression analysis of risk factors showed history of engaging in e-wastes and sex to be an independent predictor of serum TSH levels.
Igharo et al. [2020]	Cross-sectional: exposed e-waste worker (EWW) vs unexposed control group (CG), Nigeria, study period not reported	Overall *n* = 104 participants 63 male EWW 41 male residents as CG	Questionnaire Physical examination Blood samples with concentrations of HDL-, LDL-, total cholesterol (TC), triglycerides (TG) and atherogenic coefficient (AC) & -index of plasma (AIP), Castelli’s Risk Index (CRI-I & CRI-II)	Lipid profiles especially TC and LDL cholesterol were significantly higher in EWW compared to the CG. The atherogenic indices such as AC, CRI-I & CRI-II in EWW were significantly higher than in the CG.
Zhao et al. [2021]	Cross-sectional: exposed e-waste worker (EWW), southeast of China, 2018	76 EWW (35 females, 41 males)	Questionnaire incl. Demographic and work information Blood samples with concentrations of THs, TC, TG	Biochemical parameters of the EWW such as TC, TG, serum fat content, TSH, FT3, FT4 are only listed in the supplementary material without reference values, control group or textual categorisation.
Igharo et al. [2018]	Cross-sectional: exposed e-waste worker (EWW) vs unexposed control group (CG), Nigeria, 2014–2016 (some aspects were concluded in 2017)	Overall n = 104 participants 63 male EWW 41 male adults as CG	Serum samples with levels of fertility hormones LH, FSH, Testosterone (TESTO), Prolactin (PROL), Progesterone (PROG), Oestrogen (EST), Inhibin (INH)	Levels of male fertility hormones such as testosterone, progesterone, LH, FSH, prolactin and oestrogen were significantly lower, while inhibin was significantly higher in EWW compared to the CG.
Wang, Y et al. [2018]	Cross-sectional: exposed resident e-waste worker (EWW) vs unexposed control group (CG), China, study period not reported	Overall *n* = 267 participants 146 male EWW 121 males as CG	Questionnaire incl. Socio-demographics, information about exposure duration & sexual abstinence Semen samples: assessment of motility	Analysis of semen quality showed significantly lower volume of semen and total number of sperm, while the motility rate was less and the abnormality rate higher in EWW compared to controls. A multivariate, logistic regression analysis of risk factors for sperm motility rate, abnormality rate and total sperm count showed that exposure duration was a predominant risk factor.

Wang, H. et al. [38] included a third group of residents with environmental exposure, but without direct exposure through work in the informal e-waste sector, additionally to the e-waste worker and the control group of completely unexposed individuals. Significantly lower serum T3, fT3 and fT4 levels in e-waste workers and residents were found compared to the control group (*p* < 0.001) [38].

Yuan et al. reported significantly higher median level of serum TSH in e-waste workers [41]. A stepwise multivariate logistic regression analysis proofed previous exposure to e-waste and gender to be independent statistically significant predictors of serum TSH levels [41].

Focusing on lipid metabolism, Igharo et al. [32] investigated the lipid profile and atherogenic indices of e-waste workers in Nigeria. In comparison to the control group, the results of the lipid profile showed a significant increase in both total cholesterol and LDL cholesterol among e-waste workers. Notably, Atherogenic coefficient (AC), Castelli’s Risk index I and II (CRI-I and CRI-II) were significantly increased in e-waste workers [32].

They also examined serum samples from male e-waste workers for different fertility hormones. These hormones such as LH, FSH, Testosterone, Prolactin, Progesterone and Oestrogen were significantly lower in the serum of e-waste workers when compared to the control group while Inhibin was significantly elevated [19].

The male reproductive health of male e-waste workers was also analysed by Wang, Y et al. [39] and found to be negatively affected in terms of sperm quality. Sperm volume, number and motility were inversely proportional to the duration of handling e-waste and significantly lower in the e-waste workers than in the control group. Wang et al. identified exposure time, total polychlorinated biphenyls (PCBs), malondialdehyde (MDA) and Pb as predominant risk factors for semen quality [39].

#### Respiratory tract

Two studies focused especially on respiratory health in e-waste workers [18, 33] (Table 3). In their longitudinal cohort study, Nti et al. measured the effects of particulate matter exposure on the lung function of 207 study participants using spirometry in Ghana. The regression analysis showed a significant change only in the PM10, PM2.5–10 fraction and the lung function parameter FEF25–75 [18]. Kuntawee and colleagues conducted a nested case-control study with asthmatic and non-asthmatic people from an e-waste recycling site and a control area. They couldn’t associate personal characteristics and occupational factors to asthma, but ‘years of work’, showed a statistically significant association to a higher likelihood of asthma [33].

**Table 3:** Respiratory health outcomes

	Study design, exposure, setting, time	Population/ participants	Measurements, Examination	Health outcome
Respiratory tract				
Nti et al. [2020]	Longitudinal cohort study: exposed e-waste worker (EWW) vs unexposed control group (CG), Ghana, March 2017–November 2018	Overall *n* = 207 participants 142 male EWW *N* = 64/65 male adults as CG	Questionnaire incl. General, medical- & socio-demographic information Lung function measurements: Spirometry (FEV1, FVC, FEV1/FVC, PEF, FEF25–75)	The regression analysis showed a significant percent change for the lung function parameters FEF25–75 between e-waste workers and controls in the PM10, PM2.5–10 fraction.
Kuntawee et al. [2020]	Nested case-control study: exposed e-waste worker (EWW) vs unexposed control group (CG), Thailand, May–July 2017	Overall *n* = 102 participants/51 subject pairs (asthmatic & non-asthmatic) 84 EWW (49 females, 35 males) 18 residents as CG	Questionnaire incl. Lifestyle factors, use of PPE & socio-demographics Blood & urine samples	Years of work showed a statistically significant association to a higher likelihood of asthma in e-waste workers compared to controls using chi-squared test.

In several studies conducted in India, Ghana and Thailand difficulties in breathing [34], as well as cough (also with sputum) [30, 34, 37], chest pain and other respiratory problems were significantly more frequently reported in e-waste workers than in controls [34].

#### Renal function

In Ghana, no significant changes in serum creatinine and eGFR were detected in a cross-sectional study at an e-waste recycling site between e-waste workers and control group [30]. (Table 4).

**Table 4:** Renal Function

	Study design, exposure, setting, time	Population/ participants	Measurements, Examination	Health outcome
Renal function				
Feldt et al. [2014]	Cross-sectional: exposed e-waste worker (EWW) vs unexposed control group (CG), Ghana, October 2011	Overall *n* = 117 participants 75 EWW (13 females, 62 males) 42 residents as CG (8 females, 34 males)	Questionnaire & interview incl. Medical-, socio-demographic information Short physical examination Urine samples	Clinical symptoms (occurring in the last 4 weeks) such as cough, chest pain and dizziness/vertigo were reported significantly more frequently in e-waste workers compared to controls.
Neitzel et al. [2020]	Cross-sectional: exposed e-waste worker (EWW), Thailand, July 2016	Overall *n* = 119/120* EWW (*n* = 58 female, *n* = 61 male) * n = differing information for overall participants	Questionnaire incl. Socio-demographic information and self-reported health status Blood & urine samples with concentrations of calcium (Ca), creatinine, metal levels (cadmium (Cd), lead (Pb), manganese (Mn); GFR, FECa% Health & anthropometic measurements	Blood levels of cadmium and lead were significantly higher in males. Regression analysis of urinary GFR and lead among informal EWW showed a significant positive correlation.

Neitzel et al. [35] performed blood tests with a focus on renal markers in e-waste workers in Thailand, where differing GFR values didn’t prove to be gender-specific significant, but they were found to be significantly correlated with lower lead and cadmium blood levels in females (Table 4). A regression analysis of GFR and lead exposure showed a significant positive correlation among informal e-waste workers [35].

#### Cardiovascular system

Concerning cardiovascular symptoms, abnormal heart beating was noted throughout various studies, [25, 34, 37, 40] (Tables 5, 8). chest pain was reported significantly more (25.3%) in e-waste worker than within the control group [30] (Tables 4, 5, 8). Adusei et al. measured hypertension across activity spaces in e-waste workers (without control group), which was most common in collectors (17.1%), followed by burners (9.1%) and dismantlers (7.7%) [22]. High blood pressure was also diagnosed among workers in other studies [25, 34] (Tables 7, 8). Diabetes, hypertension, shortness of breath and other cardiac symptoms showed no significant differences between e-waste workers and a control group in the study of Fisher et al. [31] (Table 8).

**Table 5:** Cardiovascular system outcomes

	Study design, exposure, setting, time	Population/ participants	Measurements, Examination	Health outcome
Cardiovascular system				
Burns et al. [2016]	Cross-sectional: exposed e-waste worker (EWW), Ghana, May 2014	Overall *n* = 57 male EWW	Questionnaire incl. Demographic information, self-reported health status, noise exposure, work-related stressors	The self-reported health status of the e-waste workers showed that 24,6% experienced tinnitus and 26.3% had difficulties hearing. 54.4% were always exhausted after work and 7% always suffered from shortness of breath as well as dizziness. 14% always experienced abnormal heart beating in the last 2 weeks. Diagnosed with high blood pressure were 12.3 and 3.5% with hearing loss. 95.9% were exposed to noise at work, while 35.1% were severely bothered by that. 45.6% of the workers reported to always be exposed to unfavourable physical conditions at work.

#### Hearing system

A personal noise measurement over a 24-hour period and a hearing assessment was aimed to assess the hearing capacity of e-waste workers in Agbogbloshie, Ghana. The presence of a noise notch was positive for both ears for 32%, for the right/left ear only in 20%/18% and negative for 40% of the examined EWW (Table 6) [26]. Self-reported hearing difficulties were recorded in 2 studies at 26% [25, 26] and data on self-reported exposure to noise at work varied between 84.5% [26], 87% [24] and 95.9% [25] (Table 5). Burns et al. note that 24.6% of the e-waste workers experienced tinnitus very often and 3.5% of EWW were diagnosed with hearing loss. Difficulties in hearing were furthermore self-reported by 26.3% in a study conducted in Ghana [34] (Table 8).

**Table 6:** Hearing System

	Study design, exposure, setting, time	Population/ participants	Measurements, Examination	Health outcome
Hearing system				
Carlson et al. [2021]	Cross-sectional: exposed e-waste worker (EWW), Ghana, April 2014	Overall *n* = 58 male EWW	Survey incl. Occupational history, socio-demographics, self-reported hearing status, noise exposure Blood samples Personal noise measurement, hearing & health assessment	Self-reported hearing difficulties were reported by 26% and exposure to loud noise at work by 84.5%. 70% presented a noise notch. A right ear notch at 3 kHz,4 kHz and 6 kHz were present in 13, 18 and 27% of the e-waste worker. A left ear notch at 3 kHz,4 kHz and 6 kHz were present in 13, 20 and 31% of the e-waste worker.

#### Musculoskeletal system

Acquah et al. investigated musculoskeletal disorder symptoms among EWW in Agbogbloshie using the Cornell Musculoskeletal Discomfort Questionnaire (CMDQ). 90% of the e-waste workers reported heavy load handling, as well as 79% daily lifting, long walking (53%) and carrying (77%) [20] (Table 7).

**Table 7:** Musculoskeletal System

	Study design, exposure, setting, time	Population/ participants	Measurements, Examination	Health outcome
Musculoskeletal system				
Acquah et al. [2021]	Cross-sectional: exposed e-waste worker (EWW) vs unexposed control group (CG), Ghana, January–March 2018	Overall *n* = 217 male participants 176 EWW (*n* = 73 collectors (C), *n* = 82 dismantlers (D), n = 21 burners (B)) 41 Controls (CG)	Questionnaire incl. Demographic & occupational information Cornell Musculoskeletal Discomfort Questionnaire (CMDQ) (MSD symptoms in previous 7-days)	Prevalence of musculoskeletal discomfort for each body part by job category showed significant differences between collectors, dismantlers, burners and control group for knee, lower leg and upper arm. Pain scores showed significant differences by job category for whole body pain score, lower extremities and upper extremities. Poisson regression results predicting number of body parts with discomfort based on job category and covariates were significant for collectors (vs. CG) and dismantlers (vs. CG), as well as for age and hours worked per day.
Ohajinwa et al. [2017]	Cross-sectional: exposed e-waste worker (EWW), Nigeria, May–October 2015	Overall *n* = 279 EWW (99% male) from 3 E-waste sites (Lagos, Ibadan, Aba) divided in Dismantlers (D) and Repairers (R)	Questionnaire incl. Sociodemographic information, work practices and injury occurrence	Injury prevalence among EWW in the last 1–2 weeks and 6 months were reported by 38 and 68%, while 89% reported to have ever gotten injured. With 59.5% cuts were the most prevalent. Blunt injury/contusions (16%), electric shocks (14%) and burns (10%) were also reported. Pain in the last 12 months was most prevalent in the lower back, neck, chest and shoulders. Injury occurrence per body part was highest for hand/fingers (73%).
Burns et al. [2019]	Cross-sectional: exposed e-waste worker (EWW), Ghana, May 2014	Overall *n* = 46 male EWW	Survey incl. Demographics, health related outcome &, frequency of exposure to noise Noise measurements Personal stress factors (Cohens PSS) & occupational stress score	Fair or poor health status was self-reported by 24 EWW. The mean score for perceived stress (16 highest) was 9.9 and the mean score for occupational stress (28 highest) was 18.9. A very high level of perceived noise exposure was reported by 87% of the EWW. 94% of EWW reported injuries in previous 6 months, with 7% hospitalized for worst injury. Most common injury type were cuts/lacerations/abrasions (65%) with hand/fingers (46%). Adjusted Poisson regression model was significant for perceived noise, perceived health status and perceived stress scale.
Adusei et al. [2020]	Cross-sectional: exposed e-waste worker (EWW), Ghana, study period not reported	Overall *n* = 112 male EWW divided in Collectors (C), Sorters (S), Dismantlers (D) and Burners (B)	Survey incl. Socio- demographic information, injury experience, assessment of skin conditions	Injury experiences varied in the different areas of EWW activity (%): Cuts were most common among burners (90.9%) and dismantlers (94.4%), lacerations among dismantlers (74.4%). A significant difference (*p* = 0.038) between the groups was found for abrasions (38.5%) with dismantlers as most frequently affected group.
Acquah et al. [2021]	Cross-sectional: exposed e-waste worker (EWW) vs unexposed control group (CG), Ghana, August–October 2018	Overall n = 217 male participants 176 EWW divided in 73 Collectors (C), 21 Burners (B) and 82 Dismantlers (D) 41 Controls (CG)	Questionnaire incl. Demographic information and workload CMDQ & Occupational Physical Activity Questionnaire (OPAQ)	Pain scores of EWW were significantly higher for lower (*p* = 0.051) and upper extremity (*p* = 0.012) compared to the control group. Lower, upper back and neck pain were non-significantly more prevalent in EWW compared to the controls, as were self-reported activities and exposures such as prolonged walking, standing and sitting, daily lifting, carrying, pushing and pulling, and handling heavy loads.

Aquah and colleagues [21] calculated a pain score considering the body regions with statistically significant differences between e-waste workers and a control group for the lower and upper extremity as well as for the whole body. Comparing discomfort and pain prevalence for e-waste workers across body regions, discomfort prevalence was highest in the lower back area [21, 34]. For knees, lower legs, and upper arms, chi-square tests revealed statistically significant differences in discomfort prevalence by job category, with the highest discomfort prevalence primarily among collectors [20]. (Tables 7, 8).

**Table 8:** General self-reported health outcomes

	Study design, exposure, setting, time	Population/ participants	Measurements, Examination	Health outcome
General self-reported symptoms & health outcomes				
Mishra [2019]	Scoping Review: literature research (01/01/2010–01/01/2018) using 3 databases (PubMed, Web of Science, ScienceDirect)	E-waste workers from Ghana, India and Nigeria in overall 10 studies (5 quantitative cross-sectional studies, 3 qualitative studies, 1 mixed-methods study, 1 exploratory study)	Health Problems sorted in 5 categories: Physical injuries; Respiratory health problems; Skin problems; Musculoskeletal problems; Other general health problems	Physical injuries such as cuts were reported in 96% of EWWs in Ghana and 59.9% in Nigeria. In 3 other qualitative studies (Ghana and India), cuts and burns were the most common injuries. Breathing difficulties, coughing and chest pain were reported by EWWs in Ghana, India and Nigeria. Various skin problems such as fungal infections, itching, rashes, skin irritations and scars were reported by EWWs in Ghana and India. Musculoskeletal problems were a relevant helath problem for EWW. Other general health problems such as hearing loss, overweight, obesity, accidents at work were reported.
Decharat [2018]	Cross-sectional: exposed e-waste worker (EWW) vs unexposed control group (CG), Thailand, May–August 2016	Overall *n* = 79 participants (47 males, 32 females) from 25 e-waste shops 54 EWW 41 office workers Control Group (CG)	Survey incl. General information, use of personal protective equipment (PPE) Urine samples	The symptom prevalence differed by work position, and showed significantly more insomnia, muscle atrophy, weakness and headaches in EWW compared to the control group (p < 0.001).
Yohannessen et al. [2019]	Cross-sectional: 2 groups of informal e-waste worker (IEWW) vs formal EWW/ control group (CG), Chile, July–August 2017	Overall *n* = 93 participants (*n* = 24 female, *n* = 69 male) 53 informal EWW from Santiago (IEWW-S) 25 informal EWW from Temuco (IEWW-T) 15 formal EWW as Control Group (CG)	Questionnaire incl. Sociodemographic information, self-reported health injuries, stress, exposure to noise Health assessment, Cohen’s perceived stress scale, visual analogous scale (VAS) Blood and urine samples	Symptoms experienced in the past 2 weeks such as headache or dizziness, breathing problems, nausea/abdominal pain, skin rashes, fever, blood in urine or stool were non-significantly more frequently reported by informal EWWs (EWW-S & EWW-T). Abnormal heartbeat and other chronic diseases were reported significantly more frequently by the informal EWW (*p* = 0.008). Health problems that restrict work were significantly more common in the informal EWW group (IEWW-S) (*p* = 0.001). Additionally, significantly more punctured wounds (IEWW-S; *p* = 0.037) and burns/scalds (IEWW-T; *p* = 0.015) were reported compared to the control group. The hand was significantly more frequently injured in the informal EWW (*p* = 0.022) and the intensity of muscle soreness showed significant differences between the groups in the VAS (*p* = 0.044).
Fischer et al. [2020]	Comparative cross-sectional: exposed e-waste worker (EWW) vs unexposed control group (CG), Ghana, May 2019	Overall *n* = 178 participants 84 EWW (n = 2 female, *n* = 82 male) 94 bystanders as CG (*n* = 27 female, *n* = 67 male)	Questionnaire incl. Preexisting medical care, demographic- medical- and lifestyle information	EWW and CG showed no significant differences regarding symptoms and diseases such as infections, tuberculosis, malaria, diabetes, digestive problems, cough, high blood pressure and other cardiac symptoms, mental disorders, skin symptoms, shortness of breath, eye injuries and hearing loss. Red, itchy eyes, back pain (neck and back) and work-related injuries (cuts and burns) were significantly more common in the EWW compared to the control group (*p* < 0.05).
Seith et al. [2019]	Cross-sectional: exposed e-waste worker (EWW), Thailand, August 2016	Overall *n* = 130 EWW (*n* = 59 female, *n* = 71 male) from 4 villages (*n* = 25+ *n* = 39+ *n* = 22+ *n* = 45)	Questionnaire incl. Demographic information, work-related activities, self-reported health status and general symptoms Blood and urine samples	The self-report of the EWW indicated no high prevalence of the surveyed symptoms in the population regarding the frequency of their occurrence. A significant correlation between an increased risk for the prevalence of any symptoms was found for urinary nickel (*p* = 0.047).
Armah et al. [2019]	Cross-sectional: resident e-waste worker (EWW) vs residents (R) vs unexposed control group (CG), Ghana, January–March 2017	Overall *n* = 260 participants (*n* = 140 adult EWW (66% male); n = 60 adult R (9% male), *n* = 60 adults as CG (25% male)	Questionnaire for the occurrence of four disease symptoms (eye problems, skin burns, breathing difficulty and coughing) in the last month, demographic, and sociocultural information	Eye problems, skin burns, breathing problems and coughing were more prevalent in the resident and EWW group than in the control group. Residents freported eye problems and coughing; EWW reported skin burns and breathing problems. The analysis of the symptoms in 3 multivariate models showed that the residential-occupational status of the workers was a significant predictor for the occurrence of eye problems (EWW: *p* = 0.025, CG *p* = 0.013), skin burns (EWW: *p* = 0.003, CG p = 0.001) and breathing difficulties (EWW: *p* = 0.000, CG *p* = 0.005).

General body pain was identified as a major health problem within Mishra’s research [34]. In Ghanaian e-waste workers pain scores for upper extremities were significantly higher [20], as well as back pain (including neck) and work-related injuries compared to the control group [31] (Table 8). That also showed the high injury prevalence in a study carried out in Nigeria on three e-waste sites. 89% of the e-waste workers had been injured at some time and 38% in the last 1–2 weeks [36] while 7% were hospitalized [24]. With 96% [34], 59.5% [36] and 65% [24] cuts were the most common type of injury as also burns [34], while hand, or fingers were the most frequently injured body part with 73% [36] and 46% [24]. 40% of EWW in Ghana reported occupational accidents [34]. The job category as a risk factor associated with injuries occurring within 6 months was confirmed with statistical significance [36]. Adusei et al. also investigated the prevalence of skin conditions in different recycling activities. Rashes were highly frequent with 87.5 to 100% in all recycling tasks, skin peeling was most common within dismantlers (7.9%) while burns (77.3%) and scars (28.6%) were mostly found in burners [22] (Table 7).

#### General self-reported symptoms and health outcomes

General moderate or poor health was mentioned by 24.6 to 50% of e-waste workers in two studies [24, 37] (Table 8). Concerning dermal abnormalities, various skin problems with a prevalence up to 47.2% were reported among the workers [34]. Scars were noted to be very common [34], but overall skin rashes were the most reported [31, 37, 40] (incl. Fungal rashes [34]).

Seith et al. reported headache, bloody or watery stool and fever within the questioned e-waste workers [37] (with no control group) (Table S1).

In the questionnaire by Feldt et al. no statistically significant difference could be found for fever, abdominal pain, nausea or vomiting, diarrhoea, headache and other health problems [30] (Table S1). Only dizziness and vertigo were reported significantly more often by e-waste workers than by controls [30] (Table 4).

Similarly, the study conducted in Chile by Yohannessen et al., the informal workers reported considerably more and different symptoms than the control group, the differences did not prove to be significant [40]. Most of the chronic diseases studied were also only marginally more frequent among informal workers, except for the category “other chronic diseases” [40] (Table S1).

Fisher et al. surveyed e-waste workers in Ghana for infectious diseases such as tuberculosis and malaria, mental disorders, digestive problems, coughing, eye injuries and hearing loss, which showed no significant differences compared to the control group, as well. Although e-waste workers suffered significantly more from red itchy eyes [31] (Table 8).

Decharat, however found significantly more insomnia, muscle atrophy, weakness, and headache as symptoms in the previous month in Thai e-waste workers compared to controls [27] (Table 8).

Armah et al. used questionnaires to survey resident e-waste workers, resident non-e-waste workers and as controls - non-resident non-e-waste workers. Resident e-waste and non-e-waste-workers reported eye problems, skin burns, and respiratory problems more frequently compared to the control group. The residential-occupational status of was identified to be a significant predictor of the occurrence of eye problems, skin burns and respiratory problems, for which resident EWWs presented the highest risk [23] (Table 8).

The association between biomarker levels and health indicators, such as symptom prevalence or odds for poorer general health was studied by Seith et al., where Urinary nickel and lead in blood showed a significantly increased risk for any symptoms [37] (Table 8).

### Risk of Bias

A high risk of selection bias was found in our bias assessment in 24 of the 26 included studies, as mainly no detailed information on population recruitment was reported [18–32, 35–43] (Table 9). Concerning internal validity, a high risk of bias was detected in 12–15% of all included studies [18–21, 27, 32] and a possible detection bias was assessed in 19% of the included studies [19–21, 23, 36]. Overall, the risk was considered low in all studies [18–43] in terms of performance bias, attrition bias and reporting bias, which can be considered as a strength. An overall risk of bias assessment can be found in the supplemental section (S3).

**Table 9:** Risk of bias assessment

Study	Internal validity – bias	Internal validity – confounder	Perfor-mance bias	Detection bias	Attrition bias	Reporting bias	Selection bias
Igharo et al. [2018]	**high**	low	low	**high**	low	low	**high**
Armah et al. [2019]	low	low	low	**high**	low	low	**high**
Eguchi et al. [2015]	low	low	low	low	low	low	**high**
Zheng et al. [2017]	low	low	low	low	low	low	**high**
Nti et al. [2020]	low	**high**	low	low	low	low	**high**
Yuan et al. [2008]	low	low	low	low	low	low	**high**
Wang, H et al. [2010]	low	low	low	low	low	low	**high**
Kuntawee et al. [2020]	low	low	low	low	low	low	low
Wang, Y et al. [2018]	low	low	low	low	low	low	**high**
Yohannessen et al. [2019]	low	low	low	low	low	low	**high**
Fischer et al. [2020]	low	low	low	low	low	low	**high**
Burns et al. [2016]	low	low	low	low	low	low	**high**
Feldt et al. [2014]	low	low	low	low	low	low	**high**
Igharo et al. [2020]	**high**	**high**	low	low	low	low	**high**
Zhao et al. [2021]	low	low	low	low	low	low	**high**
Carlson et al. [2021]	low	low	low	low	low	low	**high**
Neitzel et al. [2020]	low	low	low	low	low	low	**high**
Acquah et al. [2021]	low	**high**	low	**high**	low	low	**high**
Eguchi et al. [2014]	low	low	low	low	low	low	**high**
Mishra [2019]	n.a.	n.a.	n.a.	low	low	low	n.a.
Ohajinwa et al. [2017]	low	low	low	**high**	low	low	**high**
Seith et al. [2019]	low	low	low	low	low	low	**high**
Adusei et al. [2020]	low	low	low	low	low	low	**high**
Burns et al. [2019]	low	low	low	low	low	low	**high**
Decharat [2018]	**high**	low	low	low	low	low	**high**
Acquah et al. [2021]	low	**high**	low	**high**	low	low	**high**
high risk count	3	4	0	5	0	0	24
**share of high risk**	**12%**	**15%**	**0**	**19%**	**0**	**0**	**92%**

## Discussion

To our best knowledge, this is the first systematic review of only direct occupational-related health effects in e-waste workers in the informal electronic waste recycling sector.

There is no doubt that the rudimentary way of recycling in the informal sector is causing risk to the human health [3, 13]. Various studies have repeatedly shown contaminations of local residents and e-waste workers at these sides with toxic metals, dioxins, and furans, PCBs, polyaromatic hydrocarbons (PAHs), per- and polyfluoroalkyl substances (PFAS), particulate matter, other air pollutants, phthalates or chemicals [30, 44, 45]. Even the soil in e-waste dismantling areas is heavily polluted from e-waste recycling activities [46]. Air, water, sediment and wildlife are highly contaminated with chemicals, toxic compounds and heavy metals [46, 47] like lead, cadmium and nickel, which are known to be neurotoxic, nephrotoxic, immunotoxin, carcinogen and genotoxic in humans [8, 11, 48].

The only included longitudinal study, in our systematic review covered a study period of 1.5 years [18]. Here, lung function parameters were described to be significantly lower, while the tables the authors referred to were missing in the supplementals. However, a clinically relevant pulmonary obstruction could not be derived from that study which might be due to the short observation period and the low participation rates, as mentioned before.

Longitudinal studies aiming to record organ malfunctions (e.g. progressive lung diseases) as well as symptom development and diseases with long latency periods, such as cancer are necessary. This would also be essential for the depiction of health effects of a mixed contamination with carcinogenic substances. So far, there has been a complete lack of studies on this, even though a strong biological plausibility of an association between work in the e-waste sector and health impairments urgently requires such research. Up until now, most studies have also failed to differentiate between the different work tasks in the informal sector, even though ‘job category’ was found to be a statistically significant risk factor [36]. Ideally, a study population should therefore be divided into several groups to prevent falsification of the measurements, determine the respective workload and hazards of the different exposures in order to implement necessary safety measures.

Since e-waste is mostly informally, unsafely and illegally recycled, with little or no attention paid to health protection and proper training, e-waste recycling workers do face a high risk of work-related injuries [1, 10, 11]. EWWs further reported significantly more symptoms in general [23, 27], such as cough, chest pain, difficulty breathing, abnormal heartbeat, or dizziness [21, 23, 25, 27, 30, 31, 34, 37, 40]. However, these symptoms are largely unspecific and can be caused by a wide range of diseases, circumstances, or pre-existing conditions as well as the challenging environment the workers’ are faced with. Although it is not possible to establish a causal relationship, but an association to working in the e-waste sector and impaired workers’ health is likely.

It must be considered that the low safety standards and hardly any knowledge of hazardous substances as well as little to no use of personal protective equipment (PPE) can lead to a significant likelihood of work-related impairments in the informal e-waste business in low- or middle-income countries [7]. Medical care is often only partially accessible to workers, in particular when it comes to occupational health. Therefore, it can be assumed that both occupational and non-occupational disorders receive inadequate medical attention [8, 49, 50]. Several authors therefore point to the need for occupational health measures and risk reduction through improved working practices, even though no political recommendations were made [18, 31, 38–40].

The control of transboundary movements of hazardous waste and its disposal is regulated through international agreements such as the Basel Convention of 1989 and national laws like the Resource Conservation and Recovery Act in the US. The Basel Convention, which has 182 countries and the European Union as parties, prohibits the export of e-waste without the consent of the exporting, transit and importing countries, especially if environmentally safe processing is not ensured in the importing country. However, it allows the export of e-waste for “ recycling “, which often results in misuse and illegal export. Currently, less than 50% of the contracting parties voluntarily report on their national e-waste management situation. Policy measures at national and international level are needed to improve the management of e-waste. This includes the increased enforcement of existing laws, the extension of producer responsibility (EPR) and the promotion of recycling processes [8, 12, 51]. For example, the formalization of e-waste recycling with the implementation of laws and regulations with focus on occupational health and safety might have a big impact on the worker’s health including measures such as proper training, technical and organizational measures as well as the use of personal protection equipment.

The *Solving the E-waste Problem* (StEP) initiative has developed guiding principles for the effective management of e-waste, including a clear legal framework and the extension of producer responsibility regarding financing collection and recycling, as well as the promotion of investments in recycling infrastructure [4, 52]. Better equipped border and harbor officials to combat the illegal trade in e-waste and stricter penalties for illegal exports are also important to ensure deterrence [4, 52]. A more sustainable management of e-waste can be achieved through a collective approach and the consistent implementation of these measures. Making users and manufacturers aware of the consequences of dealing with e-waste is essential [3, 53].

### General procedure/ methodology

As most of the included studies were retrospective and cross-sectional [19–32, 35–43], causal relations could only be derived for a limited number of symptoms and diseases, such as injuries or skin problems. Only one study had a longitudinal study design, with an observation period of less than 2 years [18]. Therefore, also here, any reliable statements on causality could not be given.

The presence of a control group was handled very differently among the included studies, while nine studies lacked a control group [22, 24–26, 35–37, 42, 43]. In addition, the composition of the control groups was very heterogeneous. Two studies used indirectly exposed participants as controls (e.g. residents/bystanders from the same area) [27, 31]. Another study used workers from the formal e-waste recycling sector as control [40]. The majority of the included studies had non-exposed controls (e.g. participants from another area with no known e-waste exposure) [18–21, 23, 28–30, 32, 33, 39, 41].

The methods used for data collecting such as blood, urine or semen samples, lung function and sound measurements, as well as the variance in diagnostic assessment and criteria of the examinations, for example frequencies asked in surveys or differing symptoms. Therefore, a comparison between all studies was only possible to a limited extend.

### Specific procedure/methodology

In some studies, the data described in the text were not verifiable because the tables referred to could not be found [18]. In other studies, contradictory data were described or not all required information was provided such as the specific n was not applicable and could not be determined with the given data [23]. Occasionally the *n* was reported differently [18, 35, 37] or data did not sum up to 100% [23]. In some cases, there was no information on the time of data collection or participant recruitment [38, 41] and partly it was hard to identify if the work area is considerable as formal or informal [33]. In some cases biomarker levels were described to be consistently associated but did not prove to be significant [37].

## Conclusion

This systematic review aimed to specifically analyze direct work-related health effects in informal e-waste workers caused by their work. However, due to inconsistent findings, weak associations or poor study quality, it has rarely been possible to establish a causal relationship between informal e-waste work and health effects. Only disorders of the musculoskeletal system and the work-related injuries could be directly attributed to the work in the informal e-waste recycling sector, as Issah et al. also described for the African continent [54]. Besides recycling related health effects, a challenge remains the sufficient recycling of rare elements what is so far not properly carried out in the informal sector. Of particular concern, however, is the lack of prospective longitudinal studies with sufficiently large study populations in this sector. These are urgently needed to assess adverse health effects and to capture diseases with long latency periods. In addition, a collective national and international political focus on e-waste disposal is needed and a formalization of the sector must be pursued. Occupational health and safety needs to be educated, implemented, and supported.

### Supplementary Information

**Additional file 1.** Supplementary materials S1 Detailed health outcome table; S2: Overall bias risk assessment table; S3: Search Term

## Data Availability

The datasets used and/or analysed during the current study are available from the corresponding author on reasonable request.
