# Supplementary material for: Bridging the knowledge gap! Health outcomes in informal e-waste workers
Source: J Occup Med Toxicol. 2024 Apr 15;19:11. doi: 10.1186/s12995-024-00410-z (PMC11017591; doi:10.1186/s12995-024-00410-z)
Supplement: Supplementary file 1 — Additional file 1. Supplementary materials S1 Detailed health outcome table; S2: Overall bias risk assessment table; S3: Search Term [file 12995_2024_410_MOESM1_ESM.docx]

***Supplementary materials:***

S1: Search term; S2: Detailed health outcome table; S3: Overall bias risk assessment table;

**S3: Search Term**

Pubmed:

((((worker* [TW] OR burner* [TW] OR collector* [TW] OR dismantler* [TW] OR occupation [TW] OR workplace [TW] OR worksite [TW] OR recycling [TW] OR “informal recycling” [TW]))

AND

((e-waste [TW] OR electric waste [TW] OR electronic waste [TW] OR “WEEE” [TW])

AND

(health [TW] OR “health effect*” [TW] OR contamination [TW] OR symptom* [TW] OR disease* [TW] OR (intoxication [TW] OR disorder [TW] OR injur* [TW]) OR mortality [TW] OR lethality [TW] OR morbidity [TW])))

AND

(english [Language] OR german [Language])

AND

(“1980” [Date – Publication] : “2021” [Date – Publication]))

Embase:

(english:la OR german:la)

AND

(worker* OR occupation OR workplace OR worksite OR burner* OR dismantler OR collector* OR recycling OR 'informal recycling')

AND

('e waste' OR 'electric waste' OR 'electronic waste' OR 'weee')

AND

(health OR 'health effect*' OR disorder OR symptom* OR disease* OR morbidity OR contamination OR intoxication OR mortality OR injur* OR lethality)

AND [1-1-1980]/sd NOT [2-11-2021]/sd AND [1980-2021]/py

Web of science:

All Fields:

worker* OR occupation OR workplace OR worksite OR burner* OR dismantler OR collector* OR recycling OR "informal recycling" (All Fields)

and

e-waste OR "electric waste" OR "electronic waste" OR "WEEE" (All Fields)

and

health OR "health effect*" OR disorder OR symptom* OR disease* OR morbidity OR contamination OR intoxication OR mortality OR injur* OR lethality (All Fields)

and

English OR German (Language)

**S2: Detailed health outcome table**

| *Autors/Study* | *Health outcome* |
| --- | --- |
|  | **Hormonal health (incl. thyroid function, lipid metabolism and fertility/reproductive systems)** |
| Eguchi et al. [2015]  (Eguchi et al. 2015) | TH-concentrations from Bui Dau: TT3 (Median): 1.2; TT4 (Median): 78; FT3 (Median): 3.3; FT4 (Median): 1.3; TSH (Median): 1.4  TH-concentrations from Duong Quang: TT3 (Median): 1.3; TT4 (Median): 85; FT3 (Median): 3.5; FT4 (Median): 1.2; TSH (Median): 1.5 |
| Zheng et al. [2017]  (Zheng et al. 2017) | TH concentrations in EWW were generally within the population reference ranges: TT3: Mean: 1,86 nmol/L (male), 1.73 nmol/L (female); TT4: Mean: 99.2 nmol/L (male), 103 nmol/L (female); FT3: Mean: 4,73 nmol/L (male); 4.45 nmol/L (female); FT4: Mean: 16.67 nmol/L (male); 16.62 nmol/L (female); TSH: Mean: 1,56 nmol/L (male); 2.01 nmol/L (female) -> no significant gender differences in the TH levels, except for FT3  Multiple linear regression coefficients ß [95% CI] for significant (p<0.05) or marginal (p<0.1) association between specific circulating TH levels and OC: TT3 & BDE47: 0.161 **[0.051, 0.271],** TT3 & BDE66: 0.117 **[0.029, 0.204],** TT3 & BDE85: 0.172 [**0.056, 0.288**]; TT4 & BDE85 (OC): 0.154 [**0.033, 0.276**], TT4 & BDE66 (OC): 0.161 [**0.035,0.286**]; FT3 & PCB28 (OC): -0.035 [**-0.061, -0.009**], FT3& BDE66 (OC): 0.070 [**0.006, 0.135**], FT3 & BDE85 (OC): 0.115 [**0.028, 0.203**] |
| Wang, H et al. [2010]  (Wang et al. 2018) | THs and TSH concentrations in exposure group, residents and control group (mean +/- SD): **TSH** (μIU/ml): **EWW:** **(1.26 +/- 0.72)*,** R: (1.39 +/- 1.03), CG: (1.57 +/- 0.84); **T3** (ng/ml): **EWW: (1.06 +/- 0.25)*, R:** **(1.01 +/- 0.23)*,** CG: (1.18 +/- 0.20); T4 (μg/dl): EWW: (7.96 +/- 2.02), R: (7.42+/- 1.60), CG: (7.85 +/- 1.65); **fT3** (pg/ml): **EWW: (2.72 +/- 0.46)*, R: (2.56 +/- 0.40)*,** CG: (2.86 +/- 0.37); **fT4** (pg/ml): **EWW: (0.93 +/- 0.14)*, R: (0.86 +/- 0.17)*,** CG: (1.00 +/- 0.13)  ***(p<0.001)** |
| Eguchi, A et al. [2014]  (Eguchi et al. 2014) | Concentrations of thyroid hormones (Median; Max) in EWW: TT3*,* ${ng mL}^{-1}$*:* 1.2; **2.7 (p<0.01)*;** TT4*,* ${ng mL}^{-1}$*:* 71; 120; FT3*,* ${ng mL}^{-1}$*:* 3.3; **8.2 (p<0.05)*;** FT4*,* ${ng mL}^{-1}$*:* 1.3; 1.9; TSH*, μIU* ${mL}^{-1}$*:* 1.4; 4.6  Concentrations of thyroid hormones (Median; Max) in CG: TT3*,* ${ng mL}^{-1}$*:* 1.3; 4.8; TT4*,* ${ng mL}^{-1}$*:* 79; 130; FT3*,* ${ng mL}^{-1}$*:* 3.4; 11; FT4*,* ${ng mL}^{-1}$*:* 1.3; 1.7; TSH*, μIU* ${mL}^{-1}$*:* 1.5; 8.2  ***** Max significantly lower than rural site |
| Yuan et al. [2008]  (Yuan et al. 2008) | Concentrations of TSHs (mean +/- SD): **CG: 1.12 +/-0.5; EWW: 2.27 +/-1.9; (p=0.004)**  Multivariate logistic regression analysis of risk factors 🡪 History of engaging in e-wastes (yes (1)) as independent predictor of serum TSH levels*: Serum TSH level (1): OR [95% CI]: 6.24 [0.88-44.23]; Crude OR [95% CI]: 2.77 [0.82-9.31]; Adjusted OR** [95% CI]: 6.12 [**1.58-23.72**]  * reference (no (0)) = no engagement in e-waste recycling activities ** OR adjusted by age, sex, years of residence, body mass index, smoking and alcohol drinking |
| Igharo et al. [2020]  (Igharo et al. 2020) | Lipid profiles and atherogenic indices of EWW & CG (mean+/-SD): **TC,** $\boldsymbol{mmol L}^{\boldsymbol{-}\boldsymbol{1}}$**: EWW: 4.82+/-0.71; CG: 3.84+/-0.43 (p=0.000);** TG, ${mmol L}^{-1}$: EWW: 0.40+/-0.11; CG: 0.41+/-0.12 (p=0.965); HDL cholesterol, ${mmol L}^{-1}$: EWW: 2.09+/-0.48; CG: 22.13+/-0.35 (p=0.847); **LDL cholesterol,** $\boldsymbol{mmol L}^{\boldsymbol{-}\boldsymbol{1}}$**: EWW: 2.65+/-0.43; CG: 1.64+/-0.38 (p=0.000);** AIP: EWW: -0.74+/-0.13; CG: -0.79+/-0.15 (p=0.605); **AC: EWW: 1.85+/-0.39; CG: 1.18+/-0.19 (p=0.046); CRI-I: EWW: 2.85+/-0.39; CG: 2.18+/-0.19 (p=0.046); CRI-II: EWW: 1.77+/-0.19; CG: 1.12+/-0.18 (p=0.037)** |
| Zhao et al. [2021]  (Zhao et al. 2021) | Biochemical parameters of EWW (mean+/-SD): TC,${mmol L}^{-1}$*:* 3.63+/-0.94; TG, ${mmol L}^{-1}$*:* 1.29 +/-1.04; Serum lipid content, ${g L}^{-1}$*:* 4.96 +/-1.61; TSH*, μIU* ${mL}^{-1}$*:* 1.09 +/- 0.835; FT3*,* ${ng mL}^{-1}$*:* 2.79 +/- 0.365; FT4*,* ${\mu g dL}^{-1}$*:* 0.934 +/- 0.119 |
| Igharo et al. [2018]  (Igharo et al. 2018) | Levels of male fertility hormones in exposed and unexposed participants (EWW/CG) (mean (SD); (p-value)): **LH (IU/L): 2.62 (0.25)/ 3.66 (0.29); (p=0.009); FSH (IU/L): 3.61 (0.28)/ 4.73 (0.31); (p=0.011)**; **TESTO (ng/ml): 1.56 (0.14)/ 3.09 (0.18); (p=0.000); PROL (ng/ml): 3.14 (0.48)/ 4.87 (0.66); (p=0.035)**; **PROG (ng/ml): 0.09 (0.02)/ 0.18 (0.03); (p=0.008); EST (pmol/L): 47.25 (4.63)/ 133.84 (9.35); (p=0.000); INH (ng/L): 18.89 (1.85)/ 6.94 (1.39); (p=0.008)** |
| Wang, Y et al. [2018]  (Wang et al. 2018) | Analysis of semen quality (mean +/-SD) (p-value): **Semen Volume (ml): REWW: 1.39 +/- 0.56, CG: 2.54 +/- 0.70 (p<0.01)**; Sperm concentration (1x106ml): REWW: 47.78 +/- 18.29, CG: 44.77 +/- 18.45; **Motility rate (%): REWW: 45.01 +/- 8.93, CG: 58.48 +/- 9.32 (p<0.01)**; **Abnormality rate (%): REWW: 31.11 +/- 8.33, CG: 21.77 +/- 6.54 (p<0.01)**; **Total sperm count (1x106): REWW: 102.50 +/- 30.22, CG: 117.21 +/- 32.03; (p<0.05)**  Multivariate, logistic regression analysis of risk factors for sperm motility rate, abnormality rate and total sperm count: **Exposure duration*** OR [95% CI]; (p-value): **Motility rate: 0.4 [0.2-0.7]; (p<0.01); abnormality rate: 1.9 [1.1-3.3]; (p<0.05); total sperm count: 0.5 [0.3-0.8]; (p<0.01)**  *****Unclear if data (table 5) refers to EWW only or if it includes CG |
|  | **Respiratory tract** |
| Nti et al. [2020]  (Amoabeng Nti et al. 2020) | Percent change (%) and regression analysis **ß [95% CI]** in lung function parameters (FEV1, FVC, FEV1/FVC, PEF, FEF25-75) of the respective PM: FEV1: ß[95% CI] 🡪 PM2.5: -0.001 [-0.005, 0.004], PM10: -0.001 [-0.006, 0.004], PM2.5-10: 0.002 [-0.006, 0.009]; FVC: ß[95% CI] 🡪 PM2.5: -0.001 [-0.006, 0.003], PM10: -0.002 [-0.007, 0.003], PM2.5-10: 0.003 [-0.004, 0.010]; FEV1/FVC: ß[95% CI] 🡪 PM2.5: -0.001 [-0.004, 0.002], PM10: -0.001 [-0.004, 0.003], PM2.5-10: 0.001 [-0.004, 0.005]; PEF: ß[95% CI] 🡪PM2.5: 0.006 [-0.001, 0.012], PM10: 0.007 [-0.002, 0.017]; PM2.5-10: -0.008 [-0.020, 0.003]; **FEF25-75: ß[95% CI]🡪** PM2.5: 0.005 [-0.002, 0.012], **PM10: 0.012 [0.003, 0.021], PM2.5-10: -0.015 [-0.026, 0.003]** |
| Kuntawee et al. [2020]  (Kuntawee et al. 2020) | Association of personal & occupational characteristics and asthma (dependent variable) in study participants: ‘Current work’ related/not related to e-waste: Asthma: 26%/25%; Non asthma: 20%/31% (p= 0.233); ‘Position in e-waste activity’ in relation to asthma/non asthma: Employee: 12%/10%; Registered owner: 3%/2%; Not registered owner: 11%/8%; Not related to e-waste: 25%/31% (p=0.683); ‘**Years of work’ in relation to asthma/non asthma: 1-2.99 years: 30%/31%; 3-5.99 years: 7%/12%; >6 years: 14%/8% (p=0.035)** |
|  | **Renal function** |
| Feldt et al. [2014]  (Feldt et al. 2014) | Selected symptoms (past 4 weeks) (%) of EWW & CG: **Cough: EWW: 64%; CG: 9.5% (p<0.001)**; Shortness of breath: EWW: 1.3%; CG: 0% (p=0.45); **Chest pain: EWW: 25.3%; CG: 0% (p<0.001)**; Fever EWW: 1.3%; CG: 4.8% (p=0.26); Abdominal pain: EWW: 17.3%; CG: 11.9% (p=0.43); Nausea/vomiting: EWW: 1.3%; CG: 2.4% (p=0.68); Diarrhoea: EWW: 0%; CG: 0%; Skin eruptions/lesions: EWW: 6.7%; CG: 4.8% (p=0.68); Headaches: EWW: 46.7%; CG: 28.6% (p=0.06); **Dizziness/vertigo: EWW: 16%; CG: 0% (p=0.006)**; Loss of sensation/numbness in arms/legs: EWW: 0%; CG: 0%; Concentration difficulties: EWW: 0%; CG: 2.4% (p=0.18); Sleeping difficulties: EWW: 13.5%; CG: 4.8% (p=0.14); Other health problems: EWW: 10.7%; CG: 7.1% (p=0.53)  Renal function parameters in EWW and CG (Median IQR [p25-p75] (p-value)): Creatinine, serum [mg/dl]: EWW: 0.83 [0.73-0.91], CG: 0.85 [0.62-0.96] (p=0.88); eGFR [ml/min]: EWW: 125.00 [113.91-149.78]; CG: 127.73 [110.35-140.56]; (p=0.76) |
| Neitzel et al. [2020]  (Neitzel et al. 2020) | Comparison of renal markers (GFR (mL/min/1.73m2)) between male & female informal EWW (mean+/-SD): Men: 93.45 +/-18.2, Women: 99.67+/-26.96; (p=0.194)  Regression analysis of GFR (mL/min/1.73m2)***** and metal body burden (Pb_U_) among informal EWW (B; SE (p-value)): **Pb_U_ (μg/g Cr): 1.01; 0.30 (p=0.001)**  ***** FECa% not included in this table as it has no added diagnostic value, except for very rare diseases  Spearman´s correlation between urinary- & blood metals and GFR |
|  | **Hearing system** |
| Carlson et al. [2021]  (Carlson et al. 2021) | Daily noise measurement results (N=56 EWW) (n, %): Self-reported hearing difficulties: 15, 26%; Self-reported frequency of noise exposure at work: Very often: 49, 84.5%, (Almost) never: 2, 3.4%  Noise notch results for individual frequencies and notch indices (N=55 EWW) n (%): Presence of noise notch: None: 20 (40%), Both ears: 16 (32%), Right ear only: 10 (20%), Left ear only: 9 (18%); Right ear notch: At 3 kHz: 7 (13%), At 4 kHz: 10 (18%), At 6 kHz: 15 (27%); Left ear notch: At 3 kHz: 7 (13%), At 4 kHz: 11 (20%), At 6 kHz: 17 (31%); Notch Index (dB) (Mean; SD): NN_A_: -3.3; 9.9, NN_B_: -3.8; 9.6, NN_C_: -4.9; 9.1, NN_D_: -9; 10.3  Regression models* predicting hearing outcomes in EWW: Model 1- Outcome of 4kHz hearing threshold level (Variables: Cd, Mn, Zn, Lmax (dBA), number of times eating meat/week, years living in Agbogbloshie): Adj. R^2^: 0.39, P-Value: 1x10-4  Model 2- Outcome of 6kHz hearing threshold level (Variables: Cu, Se, Zn, work activity diversity, Lmax (dBA), years living in Agbogbloshie): Adj. R^2^: 0.39, P-Value: 1x10-4  Model 3- Outcome of notch index (NND) (Variables: As, Se, Zn, work activity diversity, L_max_ (dBA), age): Adj. R^2^: 0.21, P-Value: 0.02  * table only provides Adj. R2 and p-value of model including interaction terms |
|  | **Cardiovascular system** |
| Burns et al. [2016]  (Burns et al. 2016) | Self-reported health status in categories* (%): Experience tinnitus: Never: 22,8 %, Very often: 24,6 %; Experience exhaustion after work: Sometimes: 7 %, Always: 54.4 %; Experienced shortness of breath or difficulty in breathing in the last 2 weeks: Never: 36.8 %, Always: 7 %; Experienced dizziness over the last 2 weeks: Never: 26.3 %, Always: 7 %; Experienced heart beating abnormally over the last 2 weeks: Never: 17.5 %, Always: 14 %; Diagnosed with high blood pressure: 12.3 %; On medication: 1.8 %; Experience difficulties hearing: 26.3 %; Diagnosed with hearing loss: 3.5 %  Self-reported exposures in categories* (%): Exposed to noise at work: 95.9 %; Bothered/annoyed by loud noise at work: Not at all: 22.8 %, A great deal: 35.1 %; Exposed to noise away from work: 78.9 %; Bothered/annoyed by noise at night: Not at all: 28,1 %, A great deal: 35.1 %; Sleep affected by noise at night: Never: 24,6 %, Very often: 17.5 %; Exposed to unfavourable physical conditions at work: Never: 3.5 %; Always: 45.6 %  * This table only provides the lowest and highest frequency of each category |
|  | **Musculoskeletal system** |
| Acquah et al. [2021]  (Acquah et al. 2021a) | Prevalence (%) of musculoskeletal discomfort for each body part***** by job category (C, B, D & CG): Lower back (p=0.173): C: 67%, D: 68%, B: 52%, CG: 51%; **Knee, right (p=0.001): C: 52%, D: 28%, B: 24%, CG: 20%; Lower leg, right (p<0.001): C: 47%, D: 13%, B: 10%, CG: 15%;** Shoulder, right (p=0.599): C: 36%, D: 42%, B: 29%, CG: 32%; **Upper arm, right (p<0.001): C: 30%, D: 33%, B: 5%, CG: 2%;** Neck (p=0.894): C: 25%, D: 28%, B: 24%, CG: 22%; Upper back (p=0.656): C: 12%, D: 18%, B: 10%, CG: 15%; Wrist, right (p=0.353): C: 7%, D: 15%, B: not reported, CG: 7%; Thigh, right (p=0.333): C: 7%, D: 2%, B: 0%, CG: 7%; Forearm, right (p=0.305): C: 4%, D: not reported, B: 10%, CG: 0%; Hip, buttocks (p=0.333): C: 3%, D: 0%, B: not reported, CG: 2%  Pain scores by job category and body region depicted on an average pain score for the whole body and each of the 4 body regions: (p-value) (Mean +/-SE): **Whole body pain score (sum of 4 regions) (p<0.001): C: 83.7 +/- 10.6, D: 45.5 +/- 7.6, B: 34.0 +/- 9.1, RG: 26.4 +/-5.9; Pain Score – Lower Extremities (p<0.001): C: 38.7 +/- 50.7, D: 8.5 +/- 21.8, B: 8.0 +/- 15.4, RG: 8.5 +/-21.7; Pain Score – Upper Extremities (p=0.014): C: 24.9 +/- 33.8, D: 18.5 +/- 33.2, B: 14.5 +/- 24.1, RG: 7.3 +/- 18.5;** Pain Score – Lower Back (p=0.081): C: 13.4 +/- 1.9, D: 11.7 +/- 1.8, B: 9.0 +/- 3.4, RG: 7.2 +/- 2.0; Pain Score – Upper Back and Neck (p=0.563): C: 6.7 +/- 12.7, D: 6.8 +/- 12.9, B: 2.6 +/- 4.8, RG: 3.3 +/- 8.3  ***** Only data for the right side is presented, data sorted in descending order of discomfort prevalence  Poisson regression results predicting number of body parts with discomfort reported by participants in the past week based on job category and covariates (age, hours worked per day): Estimate, B +/- SE (p-value): **Collectors (vs. CG): 0.485+/-0.132 (p<0.001); Dismantlers (vs. CG): 0.331+/-0.128 (p=0.010);** Burners (vs. CG): 0.03+/-0.182 (p=0.985); **Age (years): -0.018+/-0.008 (p=0.014);** Years on the job: 0.008+/-0.009 (p=0.380); **Hours worked per day: 0.064+/-0.015 (p<0.001);** Days worked per week: -0.057+/-0.041 (p=0.168) |
| Ohajinwa et al. [2018]  (Ohajinwa et al. 2018) | Injury occurrence among the EWW in the timelines: Injuries in the last 1-2 weeks: 38% -> D: 25%, R: 13%; Injuries in the last 6 month: 68% -> D: 37%, R: 31%; Have ever got injury: 89% -> D: 99%, R: 80%; No injury: 11%  Type of injury (%): Cuts: 59.5%, Blunt injury/contusions: 16%, Electric shocks: 14%, Burns: 10%, Falls: 0.5%  Pain in the last 12 month (%): Lower back pain: 29%, Shoulder pain: 14%, Neck pain: 29%, Chest pain: 29%, No pain: 59%  Injury occurrence per body part (%): Hand/fingers: 73%, Leg/foot: 7%, Chest/cough: 5%, Neck/shoulder: 4%, Never got injured: 11%; Predictors of injury in 6 months -> Job designation: OR [95% CI]; (p-value): **Repairers: 2.968 [1.652- 5.334]; p=0.000** |
| Burns et al. [2019]  (Burns et al. 2019) | Self-reported health status (Mean): Excellent: 9, Very good: 15, Good: 24, Fair: 44; Poor: 9  Work characteristics, activities and exposures (N=46) (Mean +/- SD): Perceived Stress Score (0 lowest to 16 highest): 9.9 +/- 2.9; Occupational Stress Score (0 lowest to 28 highest): 18.9 +/- 5.1; How much does noise annoy you? (%): Not at all: 22, A little or great deal: 80; Perceived noise exposure (%): Less than very often: 13 %, Very often: 87 %; Impairment that limits work: 20 %  E-Waste recycling injuries and activities: Number of E-waste injuries in previous 6 month (%): 94%; Number of missed days at work: 18, 39%; Hospitalized for worst injury (%): 7%  Injury type (%): Cuts/lacerations/abrasions: 65%, Burns/scalds: 4%, Internal injury: 7%, Other: 4%  Body Part injured (%): Arm: 4%, Eyes: 4%, Foot (toes): 15%, Hand (fingers): 46%, Head: 4%, Leg: 22%  Adjusted Poisson regression models (Outcome: number of injuries) -> Adjusted (AIC=397) β; SE; (ρ): Perceived noise: 0.62; 0.14, (ρ<0.001); Perceived health status: -0.27; 0.04, (ρ<0.001); Perceived Stress Scale (PSS): 0.03; 0.004, (ρ<0.001) |
| Adusei et al. [2020]  (Adusei et al. 2020) | Injury experience among EWW-groups (activity spaces) n (%): Cuts: C: 30 (85.7%), S: 13 (81.3%), D: 37 (94.9%), B: 20 (90.9%), (p= 0.799); Lacerations: C:10 (26.3%), S:5 (31.3%), D:29 (74.4%), B:12 (54.5%), (p=0.208); Abrasions: C:1 (2.8%), S:3 (18.9%), D:15 (38.5%), B:3 (13.6%), (p= 0.038)  Skin conditions among the different EWW-groups n (%):  Rashes: C: 27 (96.4%), S: 14 (87.5%), D: 39 (100%), B: 19 (90.5%), (p= 0.275); Scars: C:3 (10.7%), S:3 (8.5%), D:4 (10.5%), B:6 (28.6%), (p = 0.201); Skin peeling: C:0, S:0, D:3 (7.9%), B:1 (4.8%), (p= 0.368); Burns - Yes: C:2 (6.3%), S:1 (6.7%), D:4 (11.4%), B:17 (77.3%), (p = 0.275); Cumulative burns: C:32 (100%), S:15 (100%), D:35 (100%), B:22 (100%); Mean scar’s/person: C: 35, S: 16, D: 36, B: 22  Hypertension across activity spaces: C:6 (17.1%), S:0 (0.0%), D:3 (7.7%), B:2 (9.1%) |
| Acquah et al. [2021]  (Acquah et al. 2021b) | Pain Scores of EWW & CG (mean +/- SD) (p-value**): Lower extremities: EWW: 20.7+/-32.6, CG: 7.3 +/- 18.5, p=0.051; Upper extremities: EWW: 21.0+/-39.1, CG: 8.5 +/- 21.7, p=0.012;** Lower back: EWW: 12.1 +/- 16.3, CG: 7.2 +/- 13.0, p=0.077; Upper back and neck: EWW: 6.2 +/- 12.1, CG: 3.3 +/- 8.3, p=0.148  Self-reported activities and exposures of EWW & CG (%): Prolonged walking: EWW: 53%, CG: 16%; Daily lifting: EWW: 79%, CG: 39%; Carrying: EWW: 77%, CG: 29%; Pushing and pulling: EWW: 42%, CG: 12%; Heavy load handling: EWW: 90%, CG: 42%; Prolonged standing: EWW: 22%, CG: 24%; Sitting: EWW: 32%, CG: 33% |
|  | **General self-reported symptoms & health outcomes** |
| Mishra [2019]  (Mishra 2019) | Health Problems of EWW categorized in 5 groups across studies and countries: 1) Physical injuries: (Ghana) Cuts: 96%; (Nigeria) Cuts: 59.9%; in 3 qualitative studies (Ghana and India) cuts and burns were the most common injuries; Electric shocks: 14%; (Ghana) Eye injuries: 5.7%  2) Respiratory problems (Ghana (3 studies)): Difficulty in breathing: 15.8%, Cough: 4.6%, Chest pain: % not reported, Other respiratory problems: 4.6%; (India) Cough and Cough with sputum; (Nigeria) Chest pain: 5%  3) Skin problems: (Ghana) Overall various skin abnormalities: 47.2% (including fungal rashes: 25.3% and itching/rashes: 4.7%) -> various skin problems and scars were the most common (Ghana); (India) Skin irritation  4) Musculoskeletal problems: General body pain as a prominent health issue in 4 studies; (Nigeria) (low) back pain: 29% of EWW in last 12 month  5) Other general health problems: (Ghana (2 studies)) Difficulty in hearing: 26.3% (self-reported), % not reported in second study (clinically screened); High blood pressure: % not reported; Overweight: 25,3% (study 1), 14.8 % (study 2); Obesity: 2,3% (study 1), 33.2 % (study 2); Occupational accidents: 40%; Moderate to high level of stress, with abnormal heartbeat and dizziness(self-reported): % not reported; Stomach ache: % not reported; Headache: % not reported; Nausea: % not reported; Burning eyes: % not reported |
| Decharat [2018]  (Decharat 2018) | Prevalence of symptoms in the preceding 3 months differentiated by work position n (%): **Insomnia: EWW: 34 (63.0%), CG: 3 (12.0%), (p <0.001)**  **Muscle atrophy: EWW: 26 (48.1%), CG: 3 (12.0%), (p <0.001); Weakness: EWW: 15 (27.8%), CG: 4 (16.0%), (p <0.001); Headaches: EWW: 14 (25.9%), CG: 2 (8.0%), (p <0.001)** |
| Yohannessen et al. [2019]  (Yohannessen et al. 2019) | Self-reported health characteristics of the study population by job, type, and site: Overall health (%): Excellent: IEWW-S: 3.8%, IEWW-T: 4%, CG: 0%; Poor: IEWW-S: 9.4%, IEWW-T: 0%, CG: 0%, (p=0.701)  Symptoms in the last 2 weeks (occasionally, always, or frequently) (%): Headache or dizziness: IEWW-S: 51%, IEWW-T: 48%, CG: 33%, (p=0.674); Abnormal heart beating: **IEWW-S: 45%, IEWW-T: 12%, CG: 6.7%, (p=0.008);** Breathing problems: IEWW-S: 32%, IEWW-T: 16%, CG: 13%, (p=0.288); Nausea or stomach ache: IEWW-S: 25%, IEWW-T: 24%, CG: 20%, (p=0.962); Skin rashes: IEWW-S: 15%, IEWW-T: 20%, CG: 13%, (p=0.842); Loose or watery stools: IEWW-S: 17%, IEWW-T: 12%, CG: 13%, (p=0.408); Fever: IEWW-S: 11%, IEWW-T: 12%, CG: 0%, (p=0.625); Shaking or tremors: IEWW-S: 7.2%, IEWW-T: 8%, CG: 0%, (p=0.741); Blood in urine: IEWW-S: 5.7%, IEWW-T: 0%, CG: 0%, (p=0.674); Blood in stool: IEWW-S: 1.9%, IEWW-T: 4%, CG: 0%, (p=0.479)  Chronic diseases (%): None: IEWW-S: 42%, IEWW-T: 56%, CG: 73%, (p=0.074); High blood pressure: IEWW-S: 26%, IEWW-T: 32%, CG: 13%, (p=0.421); Diabetes mellitus: IEWW-S: 15%, IEWW-T: 20%, CG: 0%, (p=0.197); Asthma: IEWW-S: 5.7%, IEWW-T: 4%, CG: 6.7%, (p=0.927); Heart disease: IEWW-S: 9.4%, IEWW-T: 0%, CG: 0%, (p=0.136); Stroke: IEWW-S: 3.8%, IEWW-T: 4%, CG: 6.7%, (p=0.885); Kidney disease: IEWW-S: 1.9%, IEWW-T: 4%, CG: 6.7%, (p=0.631); Liver disease: IEWW-S: 0%, IEWW-T: 4%, CG: 0%, (p=0.074); Other: **IEWW-S: 30%**, **IEWW-T: 8%**, **CG: 0%**, **(p=0.008)**  Health problems that limit work (%): **IEWW-S: 28%**, **IEWW-T: 0%**, **CG: 0%**, **(p=0.001);** Unintentional weight loss last year (%): IEWW-S: 25%, IEWW-T: 8%, CG: 40%, (p=0.056)  Self-reported injuries in the study population by job, type, and site 🡪 Type of injury (%) (p-value): Cuts/lacerations: IEWW-S: 38%, IEWW-T: 28%, CG: 13%, (p=0.182); Contusions/abrasions: IEWW-S: 13%, IEWW-T: 16%, CG: 27%, (p=0.457); **Punctured wounds: IEWW-S: 15%**, **IEWW-T: 0%**, **CG: 0%**, **(p=0.037);** Sprains/strains: IEWW-S: 1.9%, IEWW-T: 8%, CG: 6.7%, (p=4.10); **Burns/scalds: IEWW-S: 0%**, **IEWW-T: 12%**, **CG: 0%**, **(p=0.015);** Fractures: IEWW-S: 1.9%, IEWW-T: 0%, CG: 0%, (p=0.683); Other: IEWW-S: 13%, IEWW-T: 28%, CG: 27%, (p=0.224)  Body part(s) injured (%) (p-value): **Hand: IEWW-S: 42%**, **IEWW-T: 48%**, **CG: 6.7%**, **(p=0.022);** Foot/lower leg: IEWW-S: 21%, IEWW-T: 8%, CG: 13%, (p=0.564); Hip: IEWW-S: 3.8%, IEWW-T: 4%, CG: 6.7%, (p=0.885); Other: IEWW-S: 11%, IEWW-T: 28%, CG: 33%, (p=0.072)  Pain in hands/wrists after e-waste working (%) (p-value): IEWW-S: 51%, IEWW-T: 44%, CG: 60%, (p=0.778); Intensity of pain in hands/wrists (mean +/- SD) (p-value): IEWW-S: 5.0 +/- 2.1, IEWW-T: 4.9 +/- 1.8, CG: 4.6 +/-1.8, (p=0.913); Muscle soreness from sitting in same position (%) (p-value): IEWW-S: 68%, IEWW-T: 48%, CG: 60%, (p=0.260); **Intensity of muscle soreness (mean +/- SD); (p-value): IEWW-S: 5.4 +/- 2.1**, **IEWW-T: 4.8 +/- 2.1**, **CG: 3.4 +/- 1.7**, **(p=0.044)** |
| Fischer et al. [2020]  (Fischer et al. 2020) | Selected symptoms and diseases of EWW and CG on Agbogbloshie scrap yard (%): Infections: EWW: 4.8%; CG: 6.4%; Tuberculosis: EWW: 2.4%, CG: 5.3%; Malaria (last 12 month): EWW: 79.5%, CG: 74.7%; Diabetes: EWW: 1.2%, CG: 1.1%; Digestive problems: EWW: 67.9%, CG: 58.5%; Hypertension: EWW: 17.9%, CG: 14.0%; Other cardiac symptoms: EWW: 26.5%, CG: 32.3%; Cough: EWW: 63.9%, CG: 64.5%; Mental disorders: EWW: 45.2%, CG: 32.3%; Skin symptoms: EWW: 43.4%, CG: 30.9%; Shortness of breath: EWW: 30.1%, CG: 25.8%; **Red itchy eyes: EWW: 67.9%, CG: 51.6%;** Eye injuries: EWW: 28.6%, CG: 19.4%; Hearing loss: EWW: 16.7%, CG: 15.1%; **Back pain (neck and back): EWW: 91.6%, CG: 79.6%; Work-related injuries (cuts and burns): EWW: 75.0%, CG: 42.6%** |
| Seith et al. [2019]  (Seith et al. 2019) | Participant characteristics (n) %: General health: Poor health: (32) 24.6%, Good health: (64) 49.2%, Excellent health: (34) 26.2%; Symptom prevalence: No symptoms: (62) 49.2%, Occasional to always: (13) 56.5%  Specific symptom prevalence EWW (%): Rash: Rarely or never: 73.0%, Occasionally, always or frequently: 27.0%; Headache: Rarely or never: 73.8%, Occasionally, always or frequently: 26.2%; Blood in urine: Rarely or never: 100%, Occasionally, always or frequently: 0%; Blood in stool: Rarely or never: 97.6%, Occasionally, always or frequently: 2.4%; Cough: Rarely or never: 82.5%, Occasionally, always or frequently: 17.5%; Abnormal heartbeat: Rarely or never: 92.9%, Occasionally, always or frequently: 7.1%  Watery stool: Rarely or never: 94.4%, Occasionally, always or frequently: 5.6%; Fever: Rarely or never: 88.9%, Occasionally, always or frequently: 11.1%  **Prevalence of any Symptoms (OR [95% CI], (p-value): Ni_Urin_ (μg/g Cr): 1.7 [1.2-2.2], (p=0.047)**  Odds of poorer general health (OR [95% CI], (p-value)): Zn_Serum_ (μg/L): 0.9 [0.7-1.2], (p=0.507); Cu_Serum_ (μg/L): 1.2 [0.6-1.7], (p=0.740); Ni_Urin_ (μg/g Cr): 0.8 [0.6-1.0], (p=0.001); Pb_Blood_ (μg/dL): 0.7 [0.5-0.9], (p=0.222); Cd_Blood_ (μg/L): 1.8 [-0.5-4.0], (p=0.053); Mn_Blood_ (μg/L): 1.6 [0.4-2.8], (p=0.095) |
| Armah et al. [2019]  (Armah et al. 2019) | Distribution of Health Symptoms (Four frequencies (not experienced, once in a week, twice in a week, several times in a week) were recorded in total **, this table only provides two items (not experienced, several times in a week): Eye problems: not experienced*: R: 24%, EWW: 43%, CG: 33%; several times in a week*: R: 63%, EWW: 38%, CG: 0% (unequal 100%); Skin burns: not experienced*: R: 24%, EWW: 44%, CG: 32%; several times in a week*: R: 43%, EWW: 57%, CG: 0%; Breathing difficulty: not experienced*: R: 25%, EWW: 41%, CG: 33% (unequal 100%); several times in a week*: R: 17%, EWW: 83%, CG: 0%; Coughing: not experienced*: R: 19%, EWW: 40%, CG: 41%; several times in a week*: R: 56%, EWW: 44%, CG: 0% (Percentage data without n, overall n= 260, specific n not applicable ** The 3 groups per experienced frequency are listed in a row and add up to 100%; *Measure of association (Pearson X2, Pr, Cramér’s) were calculated) Symptoms were analysed in 3 multivariate models in terms of contextual differences (OR [CI]*): Residential-occupational status: Eye problems: EWW: 2.02 [**1.094, 3.735**], CG: 0.36 [**0.162, 0.805**]; Skin burns: EWW: 1.84 [**1.226, 2.770**], CG: 0.46 [**0.290, 0.739**]; Breathing difficulty: EWW: 3.30 [**1.733, 6.267**], CG: 0.24 [**0.088, 0.655**  * Odds Ratio [95% Confidence Interval] |

**S3: Overall bias risk assessment table**

| Study | Study design | Internal validity – bias | Internal validity – confounder (selection bias) | Performance bias | Detection bias | Attrition bias | Reporting bias | Selection bias | Others |
| --- | --- | --- | --- | --- | --- | --- | --- | --- | --- |
| Igharo et al. [2018] | Cross-sectional study (XS) | high risk | low risk | low risk | high risk | low risk | low risk | high risk |  |
| Armah et al. [2019] | Cross-sectional study (XS) | low risk | low risk | low risk | high risk | low risk | low risk | high risk | specific n not reported-> Demographic Profile in categorial variables; Data does not always sum up to 100% |
| Eguchi et al. [2015] | Cross-sectional study (XS) | low risk | low risk | low risk | low risk | low risk | low risk | high risk |  |
| Zheng et al. [2017] | Cross-sectional study (XS) | low risk | low risk | low risk | low risk | low risk | low risk | high risk | no control group available |
| Nti et al. [2020] | Longitudinal cohort study | low risk | high risk | low risk | low risk | low risk | low risk | high risk | n was reported differently in control group (n=64, n=65); data collection periods were aligned to the seasons; supplementary materials -> tables and graphics are not provided, only graphical abstract available; in parts not possible to pair data to the group (EWW or CG) |
| Yuan et al. [2008] | Cross-sectional study (XS) | low risk | low risk | low risk | low risk | low risk | low risk | high risk | no information about the time of the data collection |
| Wang, H et al. [2010] | Cross-sectional study (XS) | low risk | low risk | low risk | low risk | low risk | low risk | high risk |  |
| Kuntawee et al. [2020] | Nested case-control study | low risk | low risk | low risk | low risk | low risk | low risk | low risk |  |
| Wang, Y et al. [2018] | Cross-sectional study (XS) | low risk | low risk | low risk | low risk | low risk | low risk | high risk | Recruitment over the same time not reported, but assumed |
| Yohannessen et al. [2019] | Cross-sectional study (XS) | low risk | low risk | low risk | low risk | low risk | low risk | high risk |  |
| Fischer et al. [2020] | Comperative cross-sectional study (XS) | low risk | low risk | low risk | low risk | low risk | low risk | high risk |  |
| Burns et al. [2016] | Cross-sectional study (XS) | low risk | low risk | low risk | low risk | low risk | low risk | high risk | no control group available |
| Feldt et al. [2014] | Cross-sectional study (XS) | low risk | low risk | low risk | low risk | low risk | low risk | high risk |  |
| Igharo et al. [2020] | Cross-sectional study (XS) | high risk | high risk | low risk | low risk | low risk | low risk | high risk |  |
| Zhao et al. [2021] | Cross-sectional study (XS) | low risk | low risk | low risk | low risk | low risk | low risk | high risk | no control group available |
| Carlson et al. [2021] | Cross-sectional study (XS) | low risk | low risk | low risk | low risk | low risk | low risk | high risk | no control group available |
| Neitzel et al. [2020] | Cross-sectional study (XS) | low risk | low risk | low risk | low risk | low risk | low risk | high risk | differing information for overall n (n=119/n=120); no control group available |
| Acquah et al. [2021] | Cross-sectional study (XS) | low risk | high risk | low risk | high risk | low risk | low risk | high risk | only data for the right side is presented |
| Eguchi et al. [2014] | Cross-sectional study (XS) | low risk | low risk | low risk | low risk | low risk | low risk | high risk |  |
| Mishra et al. [2019] | Scoping Review | n.a. (not applicable) | n.a. (not applicable) | n.a. (not applicable) | low risk | low risk | low risk | n.a. (not applicable) |  |
| Ohajinwa et al. [2017] | Cross-sectional study (XS) | low risk | low risk | low risk | high risk | low risk | low risk | high risk | no control group available |
| Seith et al. [2019] | Cross-sectional study (XS) | low risk | low risk | low risk | low risk | low risk | low risk | high risk | report of overall n from authors differs from aggregated n from village A-D; no control group available |
| Adusei et al. [2020] | Cross-sectional study (XS) | low risk | low risk | low risk | low risk | low risk | low risk | high risk | no control group available |
| Burns [2019] | Cross-sectional study (XS) | low risk | low risk | low risk | low risk | low risk | low risk | high risk | no control group available |
| Decharat [2018] | Cross-sectional study (XS) | high risk | low risk | low risk | low risk | low risk | low risk | high risk |  |
| Acquah et al. [2021] | Cross-sectional study (XS) | low risk | high risk | low risk | high risk | low risk | low risk | high risk |  |
